# Spatially aggregated clusters and scattered smaller loci of elevated malaria vector density and human infection prevalence in urban Dar es Salaam, Tanzania

**DOI:** 10.1186/s12936-016-1186-9

**Published:** 2016-03-01

**Authors:** Victoria M. Mwakalinga, Benn K. D. Sartorius, Yeromin P. Mlacha, Daniel F. Msellemu, Alex J. Limwagu, Zawadi D. Mageni, John M. Paliga, Nicodem J. Govella, Maureen Coetzee, Gerry F. Killeen, Stefan Dongus

**Affiliations:** School of Public Health, Faculty of Health Sciences, University of the Witwatersrand, Johannesburg, South Africa; Department of Housing and Infrastructure Planning, School of Urban and Regional Planning, Ardhi University, P.O. Box 35176, Dar es Salaam, United Republic of Tanzania; Environmental Health and Ecological Sciences Thematic Group, Ifakara Health Institute, Coordination Office, Kiko Avenue, Mikocheni, P.O. Box 78373, Dar es Salaam, United Republic of Tanzania; Discipline of Public Health Medicine, School of Nursing and Public Health, University of KwaZulu-Natal, Durban, South Africa; Wits Research Institute for Malaria, School of Pathology, Faculty of Health Sciences, University of the Witwatersrand, Johannesburg, South Africa; Vector Biology Department, Liverpool School of Tropical Medicine, Pembroke Place, Liverpool, L3 5QA UK; Department of Epidemiology and Public Health, Swiss Tropical and Public Health Institute, P.O. Box, 4002, Basel, Switzerland; University of Basel, 4001 Basel, Switzerland

**Keywords:** Hotspots, Spatial clustering, Mapping, *Plasmodium falciparum*, Malaria, *Anopheles gambiae*, Mosquito

## Abstract

**Background:**

Malaria transmission, primarily mediated by *Anopheles gambiae*, persists in Dar es Salaam (DSM) despite high coverage with bed nets, mosquito-proofed housing and larviciding. New or improved vector control strategies are required to eliminate malaria from DSM, but these will only succeed if they are delivered to the minority of locations where residual transmission actually persists. Hotspots of spatially clustered locations with elevated malaria infection prevalence or vector densities were, therefore, mapped across the city in an attempt to provide a basis for targeting supplementary interventions.

**Methods:**

Two phases of a city-wide population-weighted random sample of cross-sectional household surveys of malaria infections were complemented by two matching phases of geographically overlapping, high-resolution, longitudinal vector density surveys; spanning 2010–2013. Spatial autocorrelations were explored using Moran’s I and hotspots were detected using flexible spatial scan statistics.

**Results:**

Seven hotspots of spatially clustered elevated vector density and eight of malaria infection prevalence were detected over both phases. Only a third of vectors were collected in hotspots in phase 1 (30 %) and phase 2 (33 %). Malaria prevalence hotspots accounted for only half of malaria infections detected in phase 1 (55 %) and phase 2 (47 %). Three quarters (76 % in phase 1 and 74 % in phase 2) of survey locations with detectable vector populations were outside of hotspots. Similarly, more than half of locations with higher infection prevalence (>10 %) occurred outside of hotspots (51 % in phase 1 and 54 % in phase 2). Vector proliferation hazard (exposure to *An. gambiae*) and malaria infection risk were only very loosely associated with each other (Odds ratio (OR) [95 % Confidence Interval (CI)] = 1.56 [0.89, 1.78], P = 0.52)).

**Conclusion:**

Many small, scattered loci of local malaria transmission were haphazardly scattered across the city, so interventions targeting only currently identifiable spatially aggregated hotspots will have limited impact. Routine, spatially comprehensive, longitudinal entomological and parasitological surveillance systems, with sufficient sensitivity and spatial resolution to detect these scattered loci, are required to eliminate transmission from this typical African city. Intervention packages targeted to both loci and hotspots of transmission will need to suppress local vector proliferation, treat infected residents and provide vulnerable residents with supplementary protective measures against exposure.

## Background

In the city of Dar es Salaam, considerable progress has recently been made to ensure high coverage of standard interventions like rapid diagnostic tests (RDTs), artemisinin-based combination therapy (ACT), long-lasting insecticidal nets (LLINs) and other supplementary vector control measures, such as mosquito-proofed housing and regular application of microbial larvicides [1–5]. Nevertheless, stable residual malaria transmission persists (Msellemu et al. pers.comm. and [6]), so new or improved strategies for controlling malaria vectors [7–9] and parasites [8, 10] will be required to achieve elimination. However, even the most effective intervention measures can only be effective if they are delivered to locations where residual transmission persists [11]. Here the study describes how hotspots of spatially clustered locations with elevated malaria infection prevalence and vector densities were mapped across the city of Dar es Salaam, in an attempt to provide a basis for targeting supplementary interventions.

## Methods

### Study area

**Fig. 1:**
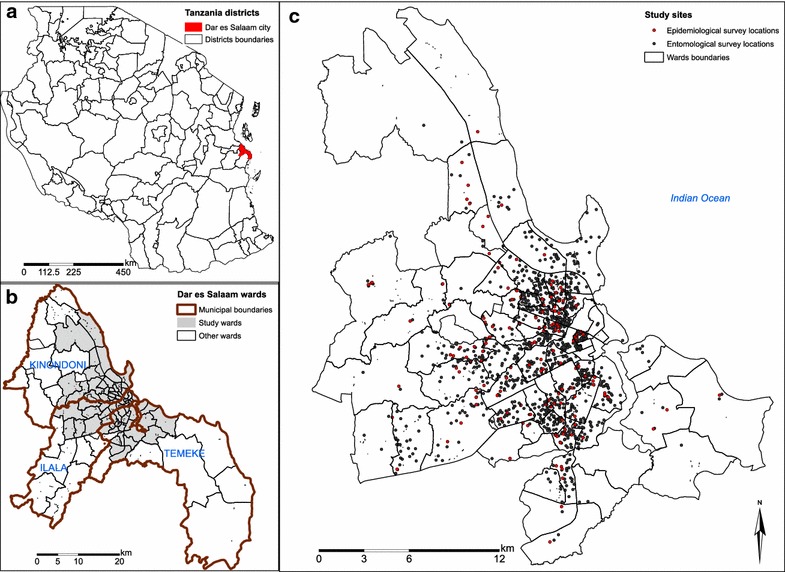
Map of the study area, epidemiological and entomological survey locations. The background is a relief shade map accessed from ESRI—online base maps on 30th Aug 2015

The study was conducted in the urban and peri-urban area of Dar es Salaam city. The city is Tanzania’s commercial and economic capital located on the shores of the Indian Ocean (Fig. 1). Dar es Salaam has 4.4 million inhabitants [12] and is amongst the world’s ten fastest growing cities [13] with an annual growth rate of 5.6 % [12]. However this growth is unplanned, resulting in about 70 % of residents living in informal settlements [14]. At the time of this study, Dar es Salaam had a mean human infection prevalence of approximately 10 %, arising from modest levels of transmission intensity, mediated by remarkably low vector densities, predominantly *Anopheles gambiae* sensu stricto [5, 15]. Administratively, the city comprises three municipalities namely Ilala, Kinondoni and Temeke and it is divided into 90 wards [12] (Fig. 1). The study area comprised 71 out of these 90 wards, covering 498.2 km^2^ of the city with approximately 3.6 million residents. Of the study wards, 15 were in phase I and all 71 wards were in phase II. The initial 15 were central wards to which larviciding was originally implemented under the Urban Malaria Control Programme (UMCP) [16] and comprehensively scaled up by the preceding operational research programme in new 56 wards [17]. In total the study covered the whole of urban and peri-urban area of Dar es Salaam city (Fig. 1). Wards are further divided into smaller neighbourhood units called *mitaa* (a Kiswahili word for *street*, written in the singular form as *mtaa*) [18]. Each *mtaa* is subdivided into ten cell units (TCUs), comprising clusters of approximately 10–20 houses, although some TCUs contain a much larger number of houses [17].

**Fig. 2:**
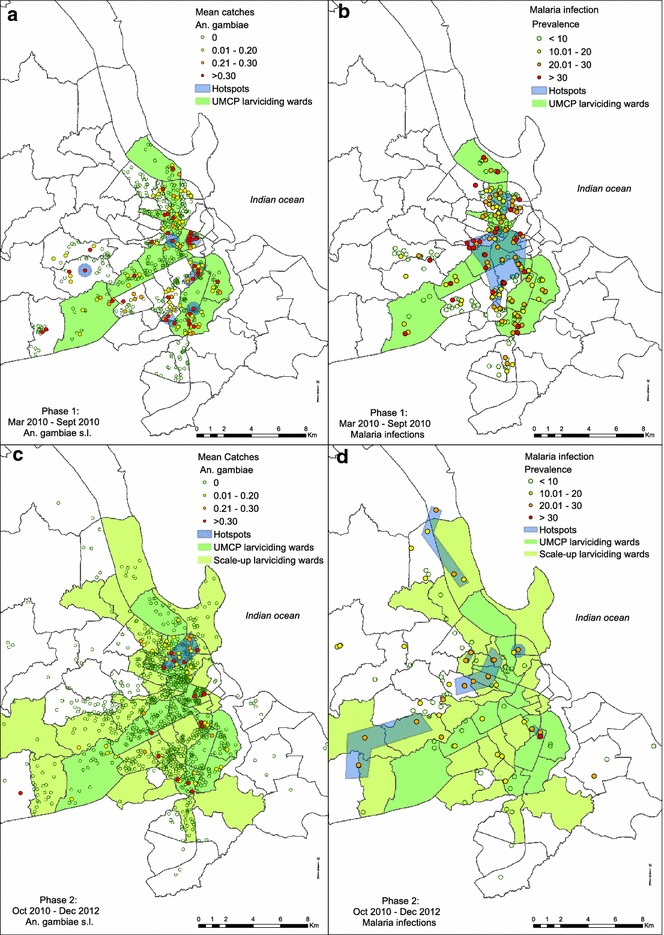
Densities of *Anopheles gambiae* vector mosquitoes and malaria infection prevalence amongst humans

With the exception of a break in activities while the programme was restructured between October and December 2010, *Bacillus thuringiensis* var. *israelensis* (*Bti*) was applied over the full course of the study, to the same 15 wards previously assessed during the operational research phase of the UMCP between 2004 and 2008, and this preventative service was scaled up to cover an additional 56 adjacent wards in January 2012 (Fig. 2). A coated granule formulation was used up to July 2011 (VectoBac^®^, Valent BioSciences Corporation), following which a pre-diluted aqueous suspension formulation (Bactivec^®^, Labiofam^®^) was used instead.

### Data collection

This study is a secondary analysis of two distinct phases of parasitological and entomological survey data (Figs. 1, 2), the collection of which is described in detail elsewhere (Msellemu et al. pers. commun). Household cross sectional and longitudinal surveys were subsequently used to collect parasitological and entomological data respectively. While two different sampling frames were used for surveying adult mosquitoes and the infection prevalence, the latter was systematically overlaid upon the former in the first phase and the former was overlaid on the latter in the second phase of surveys because mosquito surveillance sites were reduced when fund for continuously monitoring mosquito vector densities ran out [19]. Infection with blood-stage *Plasmodium falciparum* parasites was tested for using RDTs (MAL-Pf^®^, ICT Diagnostics, Cape Town, South Africa).

The total number of households in each TCU within the mapped study area [17] was enumerated by census between January 2008 and May 2010. While awaiting completion of this enumeration of all the households in the study area, for selection of a population-weighted representative sample, a first phase of purposively-sampled household surveys were conducted. This first round of household parasitological surveys was carried out from March 2010 to September 2010; consisting of 291 housing compounds (median = 7 households), 156 of which were subjected to routine collection of adult mosquitoes (Msellemu et al. pers. commun), so that this novel system [19] for monitoring vector densities at high spatial resolution could be evaluated in terms of its epidemiological predictive power.

Following completion of the household enumeration, a second phase of household surveys was begun, which sampled much smaller numbers of larger population clusters, selected randomly as index TCUs. The TCUs were weighted according to their population size and selected using a weighted sampling strategy (Msellemu et al. pers. commun). Between Oct 2010 and May 2012, a second phase of surveys was completed which encompassed a total of 86 clusters centred on index TCUs selected at random in proportion to their estimated population size (median = 392 households). Where the TCU had less than 20 consenting household heads, a neighbouring TCU was chosen at random from where the remaining number of households required to complete the sampling cluster were selected and recruited in exactly the same way. In this phase 30 of the 86 index TCUs were subjected to routine collection of adult mosquitoes. Selection of these co-surveyed areas relied upon willingness of the index TCU leaders to participate in the follow up study to monitor mosquito vector densities and also in TCUs where the environment were supportive for placement of ITT [20].

*Anopheles* mosquitoes were correspondingly collected in two different phases. The first phase lasted from March to October 2010 and consisted of 615 different sampling locations. These locations were set up within the previously studied 15 wards of the Dar es Salaam UMCP [17], which was designed to establish and evaluate systems for routine larvicide application at programmatic scales [2, 5, 16]. Following a break in funding, the second phase of entomological surveys was initiated in May 2011 and sustained through January 2013, with far greater scale-up to span a total of 1398 locations across the entire study area. The collections of adult mosquitoes were routinely conducted at each location on an approximately monthly basis, using Ifakara Tent Traps (ITT) [20] applied through a community-based system as described in detail elsewhere [19].

Throughout the study period, only modest levels of physiological resistance to pyrethroids, and behavioural resistance or resilience [15] to indoor vector control measures by biting at dawn or dusk, were observed for the *An. gambiae* complex (Chaki et al. pers.comm.). Laboratory analysis of morphologically-identified specimens of *An. gambiae* sensu lato, by polymerase chain reaction [21], confirmed that this complex was predominantly composed of the nominate sibling species *An. gambiae s.s.* [86 % (328/382)], with the small remainder being *Anopheles arabiensis* [10 % (40/382)]. *Anopheles gambiae s.s.* is the most anthropophagic and efficient sibling species from this complex, and very few *Anopheles funestus* [3 % (4/382)] or other *Anopheles* [1 % (10/382)] were caught. Vector density was therefore expressed in terms of total numbers of *An. gambiae* complex specimens caught per trap per night per TCU for subsequent analyses, and considered representative of this dominant, nominate sibling species.

The spatial references for both mosquitoes and cross-sectional sampling locations were obtained using handheld global positioning systems (GPS) at an accuracy of 5 m. These were the basis for calculating the centroids of the sampling clusters. The TCU boundaries were mapped using a participatory mapping approach [17, 18].

### Statistical analysis

Statistical analyses and data processing were performed using STATA (version 10; Statacorp). ArcGIS software (version 10; ESRI) was used to create spatial adjacency matrices, produce maps and perform exploratory spatial data analyses. FleXScan (version 3.1.2), an open source software [22] was used for the detection of spatial clusters (hotspots) in malaria infection prevalence and mosquito vector densities.

The global Moran’s I statistics (MI) [23] were employed to test for any prevailing spatial autocorrelations in mosquito vector densities and malaria infection prevalence. Spatial autocorrelation occurs when there is clustering (+ve Z-score) or dispersion (−ve Z-score) and this is significant at p ≤ 0.05 [23]. A p value > 0.05 suggests a homogeneous spatial pattern (random).

Local spatial clustering (hotspots) in mosquito densities and malaria infection prevalence were detected using the flexible spatial scan statistic (FleXScan) developed by Tango and Takahashi [24]. Flexible scan statistics create both circular and irregular shaped clusters. Since Dar es Salaam is a low transmission area, and these typical settings tend to restrict high transmission to patchier spatial scales, it was preferred to use flexible scan clustering method to enable detection of actual noncircular clusters even at highly focalized spatial scales [25]. This method identifies clusters based on a spatial weight matrix in which the detected cluster is allowed to be flexible in shape while at the same time confined within relatively small neighbourhoods of each sampling unit [25]. In developing the matrix file, spatial relationships were conceptualized based on Delaunay Triangulation, with Euclidian distance. Similar to Kulldorff’s spatial scan statistics [26], the method uses a circular moving and systematically scanning window, in which not only the whole area inside the window can be considered as a potential cluster, but also spatially connected areas inside the window, which makes it possible to detect irregular-shaped clusters [24]. The analysis parameters were set to purely spatial analysis, scanning for clusters with high rates. Hotspots were defined as spatially aggregated TCUs with significantly higher than average levels of malaria infection prevalence or *An. gambiae* mosquito densities [11]. The median values of malaria infection prevalence i.e. 12.05 % for phase 1 and 8.49 % for phase 2 were used as cut-off values in stratifying the prevalence data at TCU level, to allow analysis based on a binary logistic variable, assuming a binomial distribution for this outcome, with prevalence above the median in each phase coded as 1 and below the median coded as 0. The likelihood of clusters in mosquito densities was calculated assuming a Poisson distribution. The numerator was aggregated counts of adult female *An. gambiae* mosquitoes per TCU and the denominator was expected population of *An. gambiae* mosquitoes per TCU. Clusters of malaria infection prevalence were detected using a binomial statistical model. Statistical significance for the identification of both primary and secondary clusters was set at p < 0.05, which was explored by means of Monte Carlo replication of data sets under the null hypothesis with at least 9999 replications to ensure adequate power for defining those clusters [27, 28]. The relative risk (RR) which is the standardized risk ratio of observed mean catches of *An. gambiae* or prevalent infections over the expected mean or prevalent infections (“expected number of cases within the cluster when the null hypothesis is true, that is, when the risk is the same inside and outside the cluster”) was also presented (Table 2).

### Ethical considerations

Only participants giving informed consent were included in the study, including the mosquito catchers and the house owners where the catches took place, as well as the participants in the epidemiological cross-sectional parasite surveys. Participants found to be infected with malaria parasites during the surveys were treated with artemether-lumefantrine (Coartem^®^; Novartis Pharma AG, Basel, Switzerland) following national treatment policies and guidelines. Ethical approval for all procedures implanted in this study was obtained from the Institutional Review Board of the Ifakara Health Institute (Approval A.50), the Medical Research Coordination Committee of the Tanzanian National Institute of Medical Research (Approval NIMR/HQ/R.8a/Vol.IX/801), the Research Ethics Committee of the Liverpool School of Tropical Medicine (09.60) and the Human Research Ethics Committee (HSEC) at University of the Witwatersrand (M120835).

## Results

A total of 12,170 person nights (function of number of traps per night) of community-based ITT capture were conducted from March 2010 to January 2013. A total of 382 female *An. gambiae* were caught with an overall average catch per trap night of 0.024 (in phases I (0.064) and II (0.012). Out of 11,187 individuals who were tested for malaria infections; 1237 (11.1 %) were positive malaria cases and 9950 (88.9 %) were negative cases. The overall mean malaria prevalence was (11 %); in phase I the mean prevalence was 12.7 % (381/2754) and II was 10.8 % (854/8433).

### Spatial autocorrelation

Spatial dependences were observed in all outcome variables (Table 1). The noted positive spatial autocorrelation in both measured outcomes indicated a tendency towards clustering, meaning that spatial samples of *An. gambiae* mosquito densities or malaria prevalence that were nearby to each other in space were more similar than would be expected by random chance (Table 1).

**Table 1:** Spatial autocorrelation/dependence of mosquito densities and malaria infection prevalence in Dar es Salaam city based on global Moran’s I

Outcome variable	Phase 1: March 2010–September 2010			Phase 2: October 2010 – January 2013		
	Moran’s I coefficient	p	Mean distance between points (m)	Moran’s I coefficient	p	Mean distance between points (m)
Female *An. gambiae*	0.15	0.002	174	0.11	0.005	210
Malaria infection prevalence	0.17	0.020	208	0.34	0.011	799

p < 0.05 indicates tendency towards clustering

### Spatial clustering in *Anopheles gambiae* mosquitoes

In the first phase of the survey, six spatially aggregated clusters of elevated *An. gambiae* density were found in the central, western and southern parts of the city (Fig. 2a). These clusters were small in size and highly focalized (median = 5 survey locations, range = 1–23), with only three out of the six containing more than one survey location. Of the detected clusters, only one cluster consisting of a single location occurred outside of the 15 wards covered by larviciding (Fig. 2a), confirming that targeted selection of these wards for the initial operational research phase of the UMCP [5, 17] was appropriately planned. The clusters of relatively high *An. gambiae* density detected in this phase consisted of less than a tenth of all survey locations, but accounted for almost one third of all *An. gambiae* caught (Table 2). Nevertheless, the remaining two thirds of all *An. gambiae* were caught outside these clusters (Table 2), and three quarters of the locations where any *An. gambiae* were caught (76 %; 95/125) were haphazardly scattered outside of these clusters, across a further small minority (9 %; 57/615) of all sampling units (Fig. 2a).

**Table 2:** Spatial clusters (hotspots) of *An. gambiae* and human malaria infection prevalence in Dar es Salaam city: a flexible scan statistic was used to detect the clusters using FleXScan software Version 3.1.2 which is freely available on https://sites.google.com/site/flexscansoftware/download_e

		Phase 1: March–September 2010						Phase 2: October 2010–January 2013					
Outcome variable	Hotspot clusters	Proportion of all mosquitoes or detected infections [%(n/N)]	Proportion of all survey locations in clusters [%(n/N)]	Proportion of survey locations under larviciding [%(n/N)]	Expected mosquitoes or prevalent infections	RR	p	Proportion of all mosquitoes or detected infections [%(n/N)]	Proportion of all survey locations in clusters [%(n/N)]	Proportion of survey locations under larviciding in [%(n/N)]	Expected mosquitoes or prevalent infections	RR	p
Mean catches per trap night of female *An. gambiae*	Primary cluster	13.6 (52/382)	3.7 (23/615)	34.8 (8/23)	12.89	4.58	<0.01	32.9 (23/70)	4.8 (67/1398)	67.2 (45/67)	6.10	5.22	<0.01
	Secondary cluster 1	6 (23/382)	3.6 (22/615)	68.2 (15/22)	2.32	10.54	<0.01						
	Secondary cluster 2	3.9 (15/382)	1.5 (9/615)	33.3 (3/9)	1.27	12.33	<0.01						
	Secondary cluster 3	2.4 (9/382)	0.2 (1/615)	100 (1/1)	1.06	8.72	0.01						
	Secondary cluster 4	2.1 (8/382)	0.2 (1/615)	100(1/1)	0.85	9.67	0.02						
	Secondary cluster 5	1.6 (6/382)	0.2 (1/615)	0	0.56	10.82	0.05						
Total		29.6% (1 13/382)	9.3 (57/615)	49.1 (28/57)				32.9 (23/70)	4.8 (67/1398)	67.2 (45/67)			
Human malaria infection prevalence	Primary cluster	39.4 (148/376)	29.8 (78/261)	46.2 (36/78)	124.31	1.82	<0.01	13.4 (114/852)	5.8 (5/86)	0.6 (3/5)	53.98	2.28	<0.01
	Secondary cluster 1	15.7 (59/376)	10.0 (26/261)	50 (13/26)	18.73	2.40	<0.01	5.9 (50/852	3.5 (3/86)	100 (3/3)	21.15	2.45	<0.01
	Secondary cluster 2							7.9 (67/852)	3.5 (3/86)	0	35.04	1.99	<0.01
	Secondary cluster 3							7.3 (62/852)	4.7 (4/86)	0	33.33	1.93	<0.01
	Secondary cluster 4							9.9 (84/852)	2.3 (2/86)	0	46.22	1.91	<0.01
	Secondary cluster 5							3.2 (27/852)	1.2 (1/86)	0	10.57	2.60	<0.01
Total		55.1 (207/376)	39.8 (104/261	47.1 (49/104)				47.4 (404/852)	20.9 (18/86)	33.3 (6/18)			

Hotspots of *An. gambiae* were identified using Poisson statistical model while those of infection prevalence were detected using binomial statistical model. The significance of the cluster **(**p value) was calculated based on MonteCarlo replications set at 9999. *RR* relative risk which refers to the standardized risk ratio of observed mean catches of mosquitoes or prevalent infections (indicated by ‘n’ in a thirdcolumn) over the expected mean of mosquito densities or prevalent infections

After one year of continued larviciding and extension of the survey sampling frames, all six of the *An. gambiae* hotspots detected in phase 1 could no longer be detected (Fig. 2c), consistent with epidemiological analyses indicating larviciding succeeded in suppressing populations of these hazardous malaria vectors [3]. Despite this generally encouraging picture of success for suppression of *An. gambiae*, one new, much larger cluster emerged in phase 2 in the north-eastern parts of the city (Fig. 2c) within some of the 15 wards with long-established larvicide delivery systems. As in phase 1, the one large cluster observed in phase 2 accounted for only one third of all *An*. *gambiae* collected (Table 2). In this second phase, the majority (73.7 %; 42/57) of scattered survey-locations where any *An. gambiae* were caught occurred outside of detected hotspots of spatially-aggregated clusters of elevated vector density.

### Spatial clusters of human malaria infection prevalence

In phase 1, two spatially aggregated clusters of elevated malaria prevalence were detected (Fig. 2b). The primary cluster was a large one, located at the south of the city centre on either side of Msimbazi river valley. The only secondary cluster occurred immediately to the north of the primary cluster, in the eastern wards of Hanna Nassif, Mwananyamala and Kinondoni (Figs. 1, 2b). These clusters encompassed approximately two fifths of all survey locations and slightly more than half of all detected cases of malaria infection (Fig. 2b; Table 2). Nevertheless, the remaining majority (60 %) of locations with elevated (>10 %) prevalence, and almost half (45 %) of all detected malaria infections) occurred outside of these clusters haphazardly across disparate parts of the city (Fig. 2b; Table 2). These consisted of 50.8 % (67/132) of survey locations with higher prevalence. More than half (56.8 %, 37/65) of the survey locations in the clusters of higher malaria prevalence detected in phase 1, occurred within wards with active larviciding.

Although there was a substantial drop of malaria prevalence from the outset of phase 2, consistent with the results of detailed epidemiological analyses (Msellemu et al. pers. commun), more clusters (six) of malaria prevalence were detected (Fig. 2d). The primary cluster did not change from its previous location, but was dramatically reduced in size compared to phase 1. The remaining five clusters were in small (<1 km across) or consisted of long narrow linear pockets, distributed across peripheral parts of the city that had not previously been surveyed. In this second phase, these clusters of elevated malaria prevalence included only one fifth of all survey locations but contained almost half of all detected cases of malaria infections (Table 2). However, the remaining majority of detected infections nevertheless occurred outside of these clusters (Table 2), mostly [54.3 % (19/35)] in small, haphazardly scattered single locations with relatively high malaria prevalence (>10 %).

### Association between vector density and human infection prevalence

Although the data frames for entomological and epidemiological surveys used in this study were not entirely comparable, it is nevertheless remarkable just how dissimilar the clustering patterns are for human malaria infection prevalence and densities of the main vector in both survey phases (Fig. 2). Despite the major differences in spatial and temporal resolution of the entomological and parasitological data, it was nevertheless possible to examine their mutual association in survey locations where both types of surveys were conducted. Overall population-wide prevalence at these locations was not associated with local vector density (Odds Ratio (OR) [95 % Confidence interval (CI)] = 1.56 [0.89, 1.78], P = 0.52)). Indeed, in many locations with elevated prevalence (>10 %), no *An. gambiae* were caught despite having been surveyed at least 6 times with tent traps (17.7 % (20/113) in phase 1 and 13.2 % (10/76) in phase 2). Conversely, only a minority of co-surveyed locations (26.8 % (11/42)) in phase 1 and 37.5 % (3/8) in phase 2) where *An. gambiae* were detected had human infection prevalence exceeding 10 %.

## Discussion

The results presented here suggest it may not be possible to eliminate malaria transmission in Dar es Salaam, if interventions are only targeted to spatially clustered hotspots that could be detected with current methods for cross-sectional household surveys and longitudinal entomological surveillance. Even though two very different sampling frames were used in the two phases (Fig. 2), geographic cluster analysis consistently excluded over two thirds of all vectors caught and almost half of all malaria infections detected (Table 2). Of course, by definition, these spatial aggregations of elevated transmission account for even lower fractions of all surveyed locations (Table 2), so they do represent useful initial priority areas for geographically targeting new or improved interventions. However, these detected clusters of vector density and parasitaemia accounted for insufficient levels of aggregation to satisfy the “80–20 rule” [29] that is classically used to justify targeting of interventions to high-risk groups. So while targeting only the clusters of elevated vector density or human infection prevalence could tackle substantial portions of malaria transmission in Dar es Salaam, to achieve the ambitious goal of eliminating malaria transmission [30], or even the vector itself [31], would also require targeting of all the additional small, scattered locations where local transmission persists.

The haphazard distribution of this very sparse vector population is fully consistent with the known opportunistic oviposition habits of *An. gambiae* generally [32, 33] and in Dar es Salaam specifically [34–36]. This species can complete development from egg to adult in less than a week in almost any water body which lasts long enough, is exposed to the sun and is not heavily contaminated with organic waste [36, 37]. In order to eliminate transmission hazard, larval source management, or other supplementary vector control measures [7–9], would therefore need to not only target the obvious large clusters of high *An. gambiae* density, but also these smaller, haphazardly-distributed pockets of vector proliferation.

The scattered and sporadic distribution of malaria transmission hazard and risk in this modestly endemic setting is also consistent with previous reports from elsewhere in Africa [6, 38, 39]. Elimination of malaria from such contexts will therefore require continuous, longitudinal surveillance of both entomological and epidemiological indicators at fine geographic scales, to give dynamic, high-resolution maps of all the small, sometimes transient, hotspots of persistent transmission, so new or improved interventions can be delivered in a targeted and timely manner [6, 40]. The need for very fast response times to react to the unpredictable occurrence of unstable hot spots of largely symptomatic malaria [6] is perhaps best illustrated by the recent work from elsewhere on the east African coast, showing that targeted responses must be implemented within a month of occurrence to capture these cases in a meaningful way [38].

Remote sensing to map environmental determinants of vector proliferation hazard has achieved considerable success at local scales [41–45] but often lacks sufficient spatial resolution to detect small habitats of the type commonly used by species like *An. gambiae* [32, 33]. Locally, in this specific context, quality-assured community-based systems for using Ifakara Tent Traps have been developed for more directly monitoring and mapping transmission hazard, in terms of adult vector densities [19]. Consequently, the entomological indicators monitored here were surveyed at higher spatial (mean distance of 174 m in phase 1 and 210 m in phase 2 between survey locations) and temporal (monthly) resolution than was the epidemiological indicator. While quality-assured, community-based mosquito-trapping schemes at sentinel housing clusters centred on health facilities have been evaluated in rural Zambia [46], this study in urban Tanzania remains the only example in which such comprehensive, high-resolution coverage of an entire region has been achieved, so the general applicability of this approach will require further assessment in a greater diversity of settings.

The active cross-sectional parasitological surveys used here to assess epidemiological trends in Dar es Salaam only provided temporal snapshots of malaria dynamics, at far too coarse a geographical resolution to guide routine malaria control operations because they were distributed across a population exceeding three million people and a surface area of almost 500 km^2^, but yielded results that are nevertheless consistent with those of other studies. On the coast of Kenya, similar annual cross-sectional surveys of much smaller populations within three small rural communities allowed mapping of stable hot spots of transmission that persisted from year to year, while active longitudinal cohort surveys to detect febrile malaria enabled mapping of sporadic, unstable transmission hot spots that occur unpredictably in different locations from year to year [34]. Furthermore, a variety of other studies using intensively collected cross-sectional [47], incidence cohort [39] or health facility [38, 44, 48, 49] data within small study areas have all illustrated how hot spots ubiquitously occur at scales far smaller than 1 km.

The inadequate spatial resolution of these active cross-sectional surveys when deployed on such large, programmatically-relevant scales is particularly clearly illustrated by the results of a recently piloted passive surveillance system for mapping of malaria cases in Dar es Salaam, by tracing the home residence locations of patients reporting to health facilities with acute malaria, which demonstrated that hot spots of malaria risk in Dar es Salaam can be less than 100 m across and associations of risk with topographic wetness index can occur at similar scales (Mlacha et al. unpublished). The surprising location of the small cluster of high diagnostic positivity identified in the ward of Buguruni illustrates how mapping clusters by tracing patient’s residence from facilities data could be an effective system for detecting enigmatic, haphazardly-scattered locations of hotspots of transmission (Mlacha et al. unpublished), like the ones shown to occur all across the city by the study presented in this manuscript. Similar approaches to risk mapping using passively collected health facility data have also been successfully applied in a variety of other resource-limited contexts [38, 43–45, 48–50], so this overall strategy may offer an affordable and broadly applicable means to map malaria transmission, that yields particularly high geographic resolution in this urban context where transmission is very focal and grass-roots local government systems provide very fine-scale geographic reference points that community members can readily relate to (Mlacha et al. unpublished). Beyond scaleability and affordability, the other major advantage of facility-based mapping of malaria cases is the ability to rapidly detect sporadic, unpredictably distributed flare-ups of malaria transmission in sufficient time (≤1 month) to enable effective targeting of responsive anti-parasite interventions [38], such as focal mass drug administration or mass screening and treatment [8, 10].

As observed elsewhere [51], clusters of malaria infection prevalence only partially coincided with those of *An. gambiae* in Dar es Salaam (Fig. 2). Indeed, population-wide malaria prevalence and *An. gambiae* densities in any given location were only loosely associated to each other, in spite of malaria primarily being locally transmitted in the city, rather than imported (Msellemu et al. pers. commun). The lack of an obvious overall relationship between spatial distribution of malaria and the level of hazard presented by local vector densities may, therefore, be largely explained by differences in city’s population resilience levels, such as housing conditions, human behaviour and utilization of interventions [4, 52, 53]. Indeed more detailed epidemiological analyses of these same data confirm that high malaria risk appears to require both hazard and vulnerability: prevalence does increase dramatically with increasing local vector density among the small minority of households living in houses without protective window screening, but not among more resilient households protected by complete window screening [4]. It is, therefore, likely that intervention packages targeted to both stable and unstable hot spots of malaria transmission will need to not only reduce or eliminate the hazard represented by local vector proliferation, presumably with supplementary application of new or improved vector population abatement measures, they will also need to actively treat all local residents who are already infected with malaria [8, 10] as well as improve the resilience of the most vulnerable residents by providing them with protective measures such as bed nets [54], window screening [55] or spatial repellents [56, 57].

### Study limitations

The sampling frames for mosquito densities and human malaria infection prevalence were incomparable due to differences in geographic and temporal resolution. Similarly, the differences in sampling frames across the two phases made it impossible to compare and track temporal/spatial trends within same outcome variables. Comparison was only possible in a few sampling locations which were co-surveyed.

## Conclusions

Small scattered locations of elevated malaria transmission and densities of vector mosquitoes were haphazardly scattered across the city, so interventions targeted only to hotspots identified by geographic cluster approaches with conventional entomological and epidemiological survey methods will probably have limited impact. Routine, spatially comprehensive, longitudinal entomological, parasitological surveillance systems, with sufficient sensitivity and high spatial and temporal resolution to detect those scattered locations with elevated infections and mosquito densities, will be required to eliminate transmission from this typical contemporary city of sub-Saharan Africa. Given the loose association observed between vector proliferation hazard and malaria infection risk, intervention packages targeted to hot spots of malaria transmission will need to not only suppress local vector proliferation and treat residents who are already infected, but also provide the most vulnerable population members with supplementary protective measures against exposure.
